# *Ulva intestinalis* Protein Extracts Promote In Vitro Collagen and Hyaluronic Acid Production by Human Dermal Fibroblasts

**DOI:** 10.3390/molecules25092091

**Published:** 2020-04-30

**Authors:** Justine Bodin, Amandine Adrien, Pierre-Edouard Bodet, Delphine Dufour, Stanislas Baudouin, Thierry Maugard, Nicolas Bridiau

**Affiliations:** 1 Equipe BCBS (Biotechnologies et Chimie des Bioressources pour la Santé), La Rochelle Université, UMR CNRS 7266 LIENSs, Avenue Michel Crépeau, 17042 La Rochelle, France; justine.bodin1@univ-lr.fr (J.B.); pierreedouard.bodet@univ-lr.fr (P.-E.B.); thierry.maugard@univ-lr.fr (T.M.); 2 SEPROSYS, Séparations, Procédés, Systèmes, 12 Rue Marie-Aline Dusseau, 17000 La Rochelle, France; amandine.adrien@seprosys.com (A.A.); delphine.dufour@seprosys.com (D.D.); stanislas.baudouin@seprosys.com (S.B.)

**Keywords:** *Ulva intestinalis*, seaweed proteins, human dermal fibroblast, collagen, hyaluronic acid, anti-aging

## Abstract

With the increase in life expectancy, reducing the visible signs of skin aging has become a major issue. A reduction in collagen and hyaluronic acid synthesis by fibroblasts is a feature of skin aging. The green seaweed, *Ulva intestinalis*, is an abundant and rich source of nutrients, especially proteins and peptides. The aim of this study was to assess the potential cosmetic properties of a protein fraction from *Ulva intestinalis* (PROT-1) containing 51% of proteins and 22% of polysaccharides, and its enzymatic peptide hydrolysates on human dermal fibroblasts. PROT-1 was extracted using a patented acid- and solvent-free process (FR2998894 (B1)). The biochemical characterization and chromatographic analysis showed a main set of proteins (25 kDa). To demonstrate the anti-aging potential of PROT-1, fibroblast proliferation and collagen and hyaluronic acid production were assessed on fibroblast cell lines from donors aged 20 years (CCD-1059Sk) and 46 years (CCD-1090Sk). PROT-1 induced a significant increase in collagen and hyaluronic acid production per cell, and a reduction in cell proliferation without increasing cell mortality. These effects were reversed after protein hydrolysis of PROT-1, showing the central role of proteins in this promising anti-aging property.

## 1. Introduction

In recent years, people have started to pay more attention to skin health and beauty. The cosmetic industry is growing and many studies have focused on skin aging inhibition and delay. Wrinkles, laxity and dryness are features of aging [1]. Skin aging is a complex biological process that can be divided into two basic processes, intrinsic and extrinsic aging. Intrinsic aging is genetically inherited (genetic, hormonal and metabolic processes, cellular metabolism); extrinsic aging is due to external factors such as air pollution, toxins, nicotine consumption, lifestyle influence, chemicals and chronic light exposures, which contribute to accelerate its consequences [2]. One of the major consequences of skin aging is a reduction and alteration of extracellular matrix (ECM) components such as elastin, collagen and hyaluronic acid (HA) [3]. For this reason, maintaining collagen and HA levels in the dermis is essential to maintain a healthy skin.

HA is an ECM anionic glycosaminoglycan composed of d-glucuronic acid and *N*-acetyl-d-glucosamine, synthesized by fibroblasts and keratinocytes [4]. It plays an important role in normal dermis, of water retention and activation of tissue/fibroblast proliferation. A decrease in HA level results in impaired hydration and elastic properties of the skin [5].

Collagen is the main structural protein in the extracellular space. There are several types of collagen: in the dermis, type 1 collagen represents most of the total collagen and is involved in skin tension, elasticity and flexibility. The type 1 collagen subunit is a fibril-forming heterotrimeric protein composed of two alpha-1 chains and one alpha-2 chain which fold into a stable and highly ordered triple helix [6]. Collagen degradation is related to the expression of fibroblast-secreted matrix metalloproteinases (MMP). Indeed, during the aging process, the MMP expression increases, causing collagen degradation and reducing skin firmness [7,8].

The cosmetic industry is constantly looking for novelty, especially atypical molecules. From this point of view, the marine environment is likely to open up many new possibilities [9]. Marine macroalgae are abundant and sustainable sources of compounds for nutraceutical and cosmetic applications [10]. Among these interesting biomolecules, seaweed proteins have a high potential. The protein content of seaweeds depends on and differs according to the species, season and environmental growth conditions. Some seaweeds such as *Rhodophyta* or *Chlorophyta* contain high protein levels and are a potential renewable source to isolate biomolecules with a nutritional interest [11,12]. Green seaweeds of the *Ulva* genus can contain up to 44% of proteins [13]. *Ulva* species also contain a high level of carbohydrates and are rich sources of vitamins and minerals [14]. Green seaweeds have various biological activities, including anticoagulant, anti-aging, antimicrobial and antifungal, antioxidant, mitogenic, immunomodulatory, anti-cholesterol and many other potential activities [15,16,17]. Among the *Ulva* genus, the edible macroalga *Ulva intestinalis* contains 19.5% of proteins during summer [18]. In addition, it has been shown that a protein hydrolysate derived from *Ulva lactuca* exhibited an anti-aging activity. This protein hydrolysate, which was composed of numerous amino acids, including lysine, histidine, arginine, aspartic acid, proline, glycine, serine, glutamic acid, alanine, threonine, tyrosine, isoleucine, leucine, and phenylalanine, optimized cellular respiration, stimulated collagen production in the skin and inhibited elastase activity [19].

**Scheme 1:**
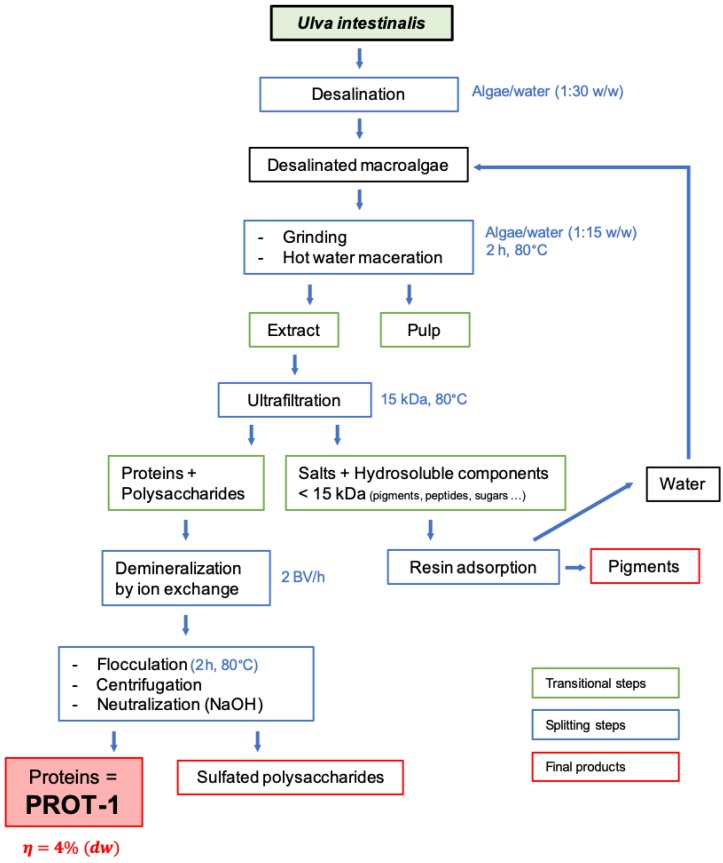
Different steps and yield (η) of the extraction procedure developed by SEPROSYS [20].

In this study, we used a previously developed acid-free and solvent-free procedure to extract and purify proteins from *Ulva intestinalis* (Scheme 1) [20]. Then, we developed a hydrolysis procedure of the obtained protein fraction (PROT-1) to produce a protein fraction of reduced molecular weight (MW), H-PROT-1, in order to assess the influence of MW on collagen and hyaluronic acid production by normal human dermal fibroblast (NHDF) cell lines from donors aged 20 and 46 years.

## 2. Results and Discussion

### 2.1. Production and Characterization of Protein Fraction (PROT-1) and Protein Hydrolysates (H-PROT-1)

*Ulva intestinalis* material was shown to contain 26 ± 2.8% (*w*/*w*_dry material_) of proteins, as determined by the Kjeldahl method, which was within the high values reported for green seaweeds of the *Ulva* genus (7–33% (*w*/*w*_dry material_)) [21].

The protein fraction PROT-1 was extracted by following the patented Seprosys process illustrated on Scheme 1 [20]. The extracted protein fraction represented 4% (*w*/*w*_dry material_) (21 g) of the desalinated dry macroalgae (on 500 g of algae). The efficiency of the aqueous and solvent-free protein extraction differs depending on the starting sample and protein accessibility. Indeed, some components such as polysaccharides may interfere with algae protein extract. The yield obtained here was in the average of the yields of protein aqueous extraction found in the literature [22].

**Figure 1:**
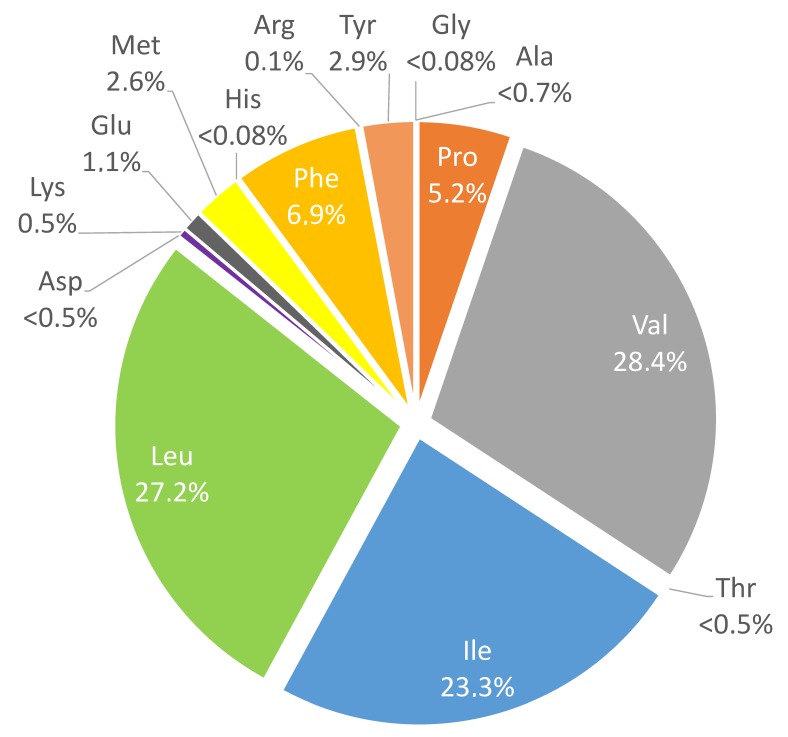
Total amino acids composition of the PROT-1 fraction determined after acid hydrolysis and UHPLC-HRMS analysis.

The total amino acid composition of PROT-1 was determined after acid hydrolysis under strong acidic conditions at 100 °C for 2 h, using UHPLC-HRMS analysis of the amino acid mixture obtained after neutralization. Figure 1 shows that the fraction was mainly composed of leucine (27.2%), isoleucine (23.3%) and valine (28.4%). According to Lewis et al., major amino acids composing *Ulva intestinalis* seaweeds are proline, methionine and aspartic acid [23], which is different from what we found. However, amino acid composition of the alga can drastically vary with respect to the season, period or geographic place of harvest, but also to its growth stage [24], which could explain these differences.

**Figure 2:**
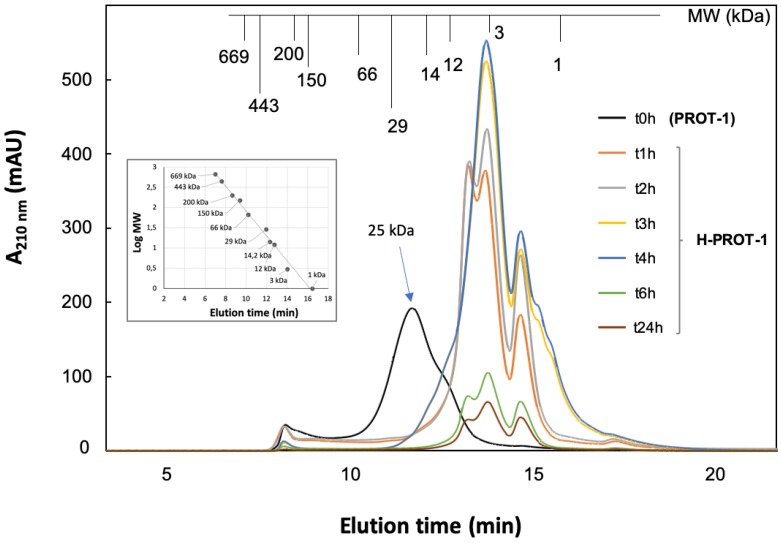
Standard curve and time-course HPLC-SEC profiles of PROT-1 enzymatic hydrolysis catalyzed by the protease preparation Flavourzyme^®^.

The PROT-1 fraction was also enzymatically-hydrolyzed by using the protease preparation Flavourzyme^®^, in order to obtain a fraction containing low MW peptides, thereafter referred as PROT-1 hydrolysates (H-PROT-1 (t2h), (t4h), (t6h), (t24h)). The enzymatic hydrolysis procedure was optimized to determine the optimal conditions (data not shown). In order to follow the time-course of hydrolysis, a monitoring method involving a separation by high-performance liquid size exclusion chromatography (HPL-SEC) was developed, and the MW of protein and peptide sets was estimated using standards of known MW. Figure 2 shows the enzymatic hydrolysis time-course of the PROT-1 fraction. The analyses showed that this fraction contained a major set of proteins with a MW of about 25 kDa, eluted at 12 min, and a set of proteins with a MW higher than 400 kDa, eluted at 7 min. During the hydrolysis, a shifting of the main peak towards higher retention times was observed, which reflected a decrease in molecular weight. For example, at t1h, two major peaks at 13.4 and 13.9 min corresponding to sets of proteins of 7.2 and 5.1 kDa, respectively, were observed. Furthermore, the profile observed after 6 h of depolymerization was probably the consequence of a profound enzymatic hydrolysis of peptides, causing the liberation of amino acids that were not or not much detected on the chromatograms, due to their very low molar extinction coefficient compared to polypeptides.

**Figure 3:**
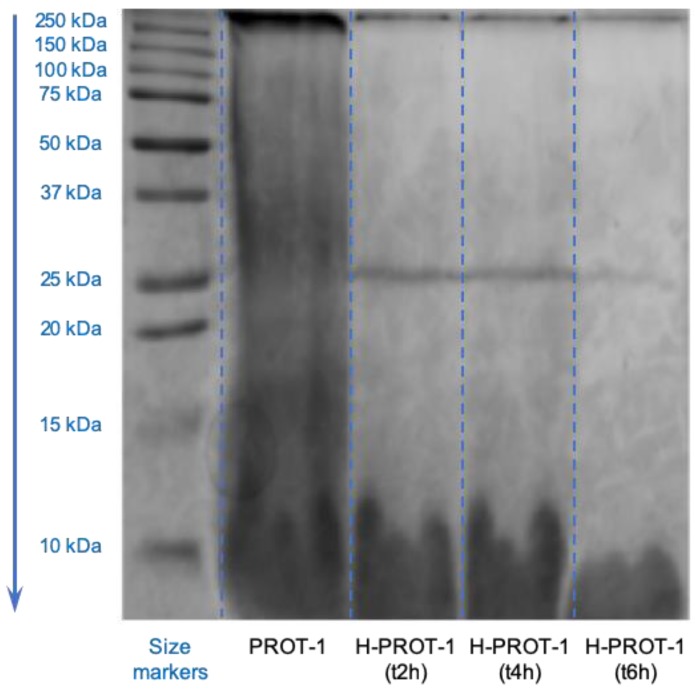
SDS-PAGE analysis of the PROT-1 fraction followed during enzymatic hydrolysis catalyzed by the protease preparation Flavourzyme^®^.

The PROT-1 fraction contained 51% of proteins and 22% of polysaccharides (Table 1). The presence of polysaccharides may be explained by two reasons: the presence of a cell wall mucilage containing polysaccharides [25] and the well-known presence of glycoproteins in algae of the *Ulva* genus [26]. The protein purity of the fraction could be improved by controlling the presence and composition of anionic and neutral polysaccharides composing the mucilage. To characterize PROT-1 and its hydrolysates obtained after enzymatic hydrolysis, all samples were analyzed using SDS-PAGE (Figure 3). Lane 1 contains the size markers and lane 2 contains PROT-1. The visible trail in lane 2 indicates a polydisperse set of proteins, very likely due to the partial hydrolysis of proteins that could occur during the extraction step under harsh conditions, especially during flocculation. This protein trail disappeared in accordance with the time-course of the enzymatic hydrolysis, showing its efficiency. The presence of a band at 25 kDa at t2h is in line with the results reported by Rouxel et al. on the existence of a water-soluble protein with a MW of 25 kDa in *Ulva intestinalis* [27]. The intensity of this band at 25 kDa and others corresponding to low MW (around 10 kDa) decreased throughout hydrolysis, which is consistent with the HPLC-SEC analyses.

**Figure 4:**
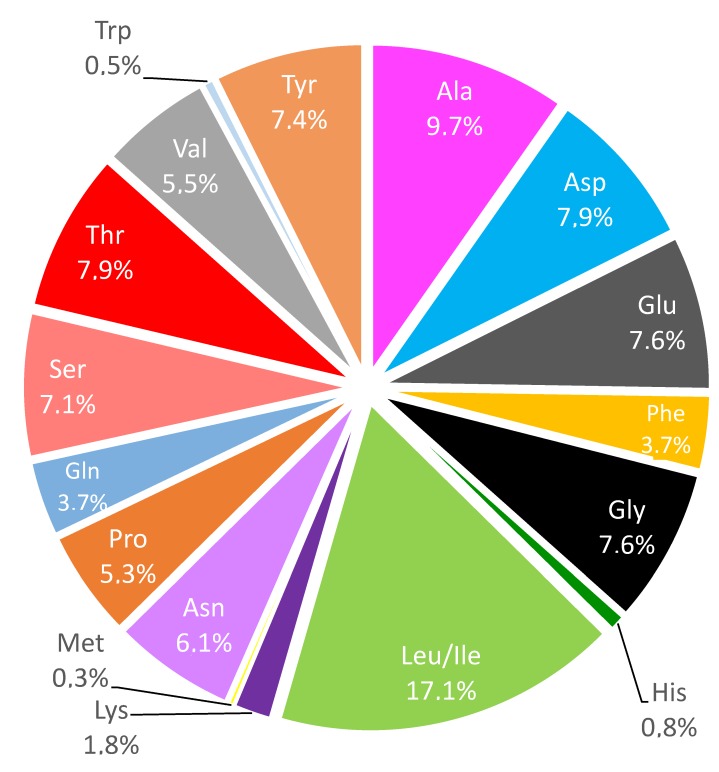
Amino acid composition of the 88 peptides identified in the H-PROT-1 (t1h) fraction by UHPLC-HRMS.

Table 2 lists the peptides that were identified in the H-PROT-1 (t1h) fraction using UHPLC-HRMS. 88 peptides were identified, more precisely 14 dipeptides, 4 tripeptides, 17 tetrapeptides, 14 pentapeptides, and 39 peptides of 6 to 15 amino acids. The amino acid composition of these 88 peptides was then determined according to their sequences and showed that these peptides were mostly composed of leucine or isoleucine (17.1%), alanine (9.7%), and glycine, glutamic acid, aspartic acid, tyrosine, threonine and serine in similar proportions, between 7.1% and 7.9% (Figure 4).

### 2.2. Assessment of PROT-1 and H-PROT-1 Cytotoxicity on NHDF

The aim of this part was to assess the cytotoxicity of the PROT-1 and H-PROT-1 fractions (t2h, t4h, t6h and t24h) on NHDF at 10, 100 and 500 µg/mL. Two fibroblast cell lines were used: one from a 20-year old woman (line 1059) and one from a 46-year old woman (line 1090). These two cell lines were used to determine whether the effects could vary according to the age of the donor skin.

**Figure 5:**
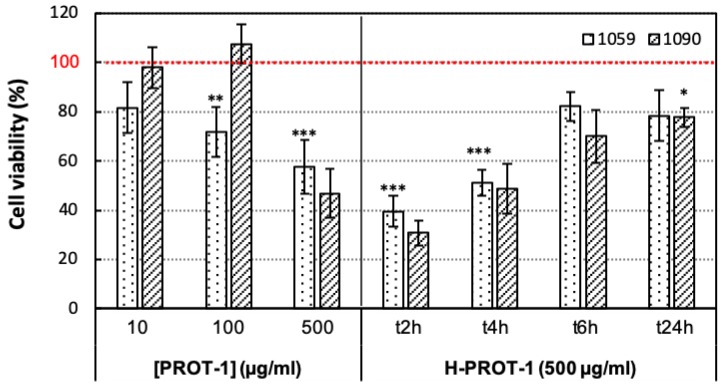
Effect of PROT-1 and H-PROT-1 on fibroblast proliferation in vitro (MTT assay). Results are expressed as the mean relative percentage of viable fibroblasts compared to the negative control (100%). Significant differences between values obtained with samples and negative control (*n* = 20) are indicated by * (*p* < 0.05), ** (*p* < 0.01) and *** (*p* < 0.001).

Figure 5 shows the effect of PROT-1 and H-PROT-1 on fibroblast viability. PROT-1 decreased fibroblast proliferation, reducing by 18%, 28% and 42% the proliferation of the 1059-cell line at 10, 100 and 500 µg/mL, respectively. The effect was less marked for the 1090-cell line as there was no effect on cell proliferation at 10 and 100 µg/mL but a decrease of 53% was observed at 500 µg/mL. Thus, a slight difference was observed between both cell lines. The 1059-cell line was rapidly affected by the extract, at a lower concentration than the 1090 cell line. The effect of the low MW peptide fractions H-PROT-1 (t2h, t4h, t6h and t24h) on fibroblast proliferation was lower compared to that of PROT-1 on both cell lines. Indeed, in the wells treated with H-PROT-1 (t24h), the cell viability reached 79% for the 1059-cell line and 78% for the 1090-cell line. These results showed that decreasing the MW of PROT-1 proteins led to a reduced effect on cell proliferation for both cell lines.

**Figure 6:**
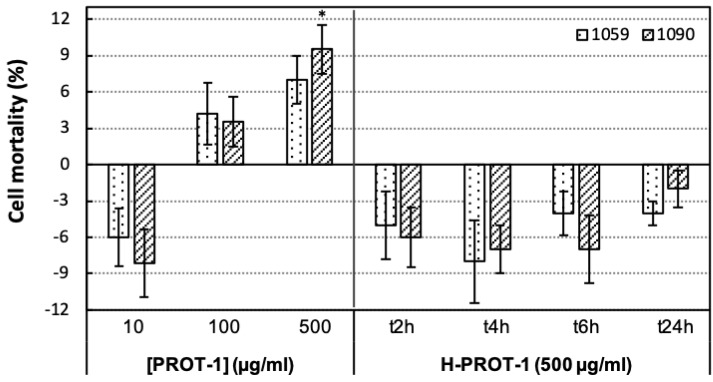
Effect of PROT-1 and H-PROT-1 on fibroblast mortality in vitro (LDH assay). Results are expressed as the mean relative percentage of living fibroblasts compared to the negative control (0%). Significant differences between values obtained with samples and negative control (*n* = 8) are indicated by * (*p* < 0.05).

Figure 6 shows the effect of the PROT-1 fraction and H-PROT-1 hydrolysates on the cell mortality by necrosis, compared to the negative control. PROT-1 had no effect on cell mortality while a slight decrease in cell mortality was observed with H-PROT-1 at t2h, t4h and t6h: −8% (cell line 1059) and −7% (cell line 1090) for example at t4h. Except for the highest concentration of PROT-1, 500 µg/mL, which led to a very slight increase in cell mortality (+7% for the 1059-cell line and +9.5% for the 1090-cell line), no deleterious effect was observed on fibroblasts.

Overall, PROT-1 decreased cell proliferation without increasing cell mortality by necrosis. Besides, we did not observe any alteration of the fibroblast morphology when checking the cells with optical microscopy. PROT-1 thus appeared to have a cytostatic effect on both cell lines in a similar way, but this effect was reduced after enzymatic protein hydrolysis. This would therefore indicate that the molecular weight or the distinctive structure of some PROT-1 proteins is related to this cytostatic effect.

### 2.3. Effect of PROT-1 and H-PROT-1 on Collagen Production

In this part of the study, the effect of the PROT-1 and H-PROT-1 fractions on collagen production was assessed. Hyaluronic acid (HA) was used as a positive control. Indeed, HA is recognized by CD44 receptors on the cell surface, which activates signaling pathways involved in the stimulation of fibroblast proliferation and collagen production [28,29].

**Figure 7:**
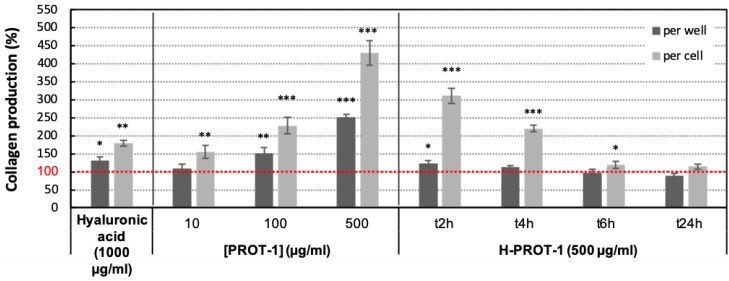
Collagen production per well and per cell by the human dermal fibroblast cell line 1059. Results are expressed as the mean relative percentage of production, compared to the negative control (100%). Significant differences between values obtained with samples and negative control (*n* = 9) are indicated by * (*p* < 0.05), ** (*p* < 0.01) and *** (*p* < 0.001).

**Figure 8:**
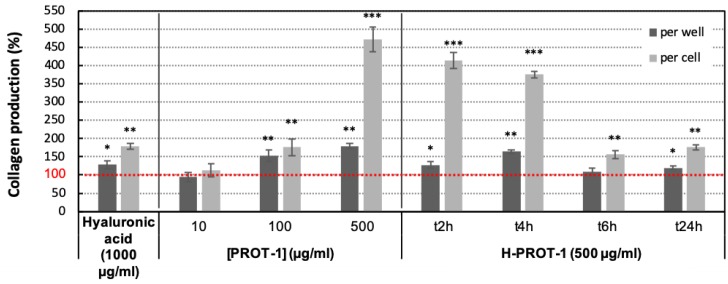
Collagen production per well and per cell by the human dermal fibroblast cell line 1090. Results are expressed as the mean relative percentage of production, compared to the negative control (100%). Significant differences between values obtained with samples and negative control (*n* = 9) are indicated by * (*p* < 0.05), ** (*p* < 0.01) and *** (*p* < 0.001).

The protein fractions showed a higher activity than the positive control (Figure 7 and Figure 8). More specifically, at a HA concentration of 1000 µg/mL, the collagen production per well was increased by 30.5% for the 1059 cell line (Figure 7) and by 28.5% for the 1090 cell line (Figure 8), while PROT-1 induced a significant dose-dependent rise in collagen production per well by 51.9% and 151.8% at 100 and 500 µg/mL, respectively, for the 1059 cell line, and by 52.5% and 79.5% at 100 and 500 µg/mL, respectively, for the 1090 cell line. However, the reduction of PROT-1 proteins MW led to a strong decrease in collagen production. Indeed, after 24 h of hydrolysis, the collagen production per well did not significantly differ from the control conditions.

We also studied the collagen production per cell. In the presence of HA at a concentration of 1000 µg/mL, the collagen production per cell increased in a similar way with both cell lines, by about 80%. The results obtained with the PROT-1 fraction were very interesting as they showed a very high dose-dependent increase in collagen production per cell, compared to the negative control: +55%, +128% and +331% for the 1059 cell line (Figure 7) and +13%, +76% and +371% for the 1090 cell line (Figure 8), at 10, 100 and 500 µg/mL, respectively. It is noteworthy, however, that this pro-collagen activity was lost when hydrolyzing proteins in the fraction PROT-1, particularly for the 1059 cell line. Indeed, the production of collagen per cell did not significantly increase in presence of H-PROT-1 at t24h. This effect seemed even closely related to the degradation of proteins in the H-PROT-1 fraction as the pro-collagen activity of the PROT-1 fraction decreased in accordance with the time-course of the enzymatic hydrolysis reaction. These results suggest that the native structure of the proteins that are responsible for the pro-collagen activity of the PROT-1 fraction is essential. The results obtained with the 1090 cell line were very similar, except that this cell line remained slightly more stimulated by the H-PROT-1 fractions.

These results are of particular interest as the pro-collagen activity of the PROT-1 and even H-PROT-1 fractions appeared to be superior to that of hyaluronic acid, which is a pro-collagen active compound usually used in anti-aging skin care cosmetic formulations [30,31]. Furthermore, this activity could be explained by the induction of a cell metabolic redirection, which would also be in accordance with the cytostatic effect of the fractions that we previously observed. It means that fibroblast moved towards collagen biosynthesis pathway(s) rather than cell growth. Indeed, fibroblasts underwent a rapid decrease in cell viability but no significant change in cell mortality and morphology. Kmail et al. showed a very similar behavior of hepatic macrophages in contact with extracts from *Asparagus aphyllus, Crataegus aronia,* and *Ephedra alata*, which induced a significant cytostatic effect on macrophage cultures, highlighted by MTT and LDH tests. These authors observed a decrease in cell viability but no cytotoxicity of these antidiabetic extracts [32].

The very significant decrease in the pro-collagen activity that was observed with the hydrolyzed protein fractions confirmed that the proteins were the molecules responsible for the collagen synthesis stimulation in PROT-1. This result was consistent with what Ko et al. highlighted when assessing the effect of a protein extract from *Ulva pertusa* on the proliferation and type I collagen synthesis of replicative senescent fibroblasts [33], as well as the pro-collagen activity of a protein extract from *Ulva lactuca*, AOSAINE^®^, developed by BiotechMarine. It was also consistent with the work of Montanari and Guglielmo, who reported a tripeptide of sequence Lys-Val-Lys, which was shown to promote collagen synthesis by human fibroblasts by 75% when associated to an *Ulva lactuca* aqueous extract [34]. On the contrary, our results differed from what the same authors found when they extracted water-soluble proteins from *Ulva lactuca* and produced a polypeptide containing the sequence Arg-Gly-Asp, after depolymerization of these proteins. This polypeptide was shown to promote collagen I production by stimulating the proliferation of fibroblasts and not collagen biosynthesis. Similarly, Honma et al. demonstrated that peptide sequences Leu-Glu-His-Ala, Leu-Asp-His-Ala or Leu-Glu-His-Ala-Phe, could promote extra-cellular collagen production by NHDF [35]. Nevertheless, the mechanisms of actions involved in the activities of these peptides or proteins remain unclear. The protein extract from *Ulva pertusa* was proved to directly inhibit MMP-1, which could explain its activity by preventing type I collagen degradation. Joe et al. reported that an extract from *Ecklonia stolonifera,* containing phlorotannins, could inhibit NF-kB or Ap-1 reporter gene expression and therefore suppress the expression of MMP-1 in NHDP, which led to the increase in collagen production [36]. A very similar activity was also shown with a peptide from *Chlorella vulgaris* [37]. At last, a pentadecapeptide from *Pyropia yezoensis* (Asp-Pro-Lys-Gly-Lys-Gln-Gln-Ala-Ile-His-Val-Ala-Pro-Ser-Phe), was shown to be able to activate the TGF-β/Smad signaling pathway, leading to increased type 1 collagen expression and upregulated transcription factor specificity protein 1 (Sp1) expression, which is reportedly involved in type 1 collagen expression [38].

None of these peptide sequences was found in the 88 peptide sequences identified in the H-PROT-1 (t1h) fraction, obtained after 1 h of PROT-1 hydrolysis by the enzyme preparation Flavourzyme^®^. Nevertheless, one tripeptide sequence Thr-Val-Asn, including a central valine and two external polar amino acids, was found in the peptides eluted at 9.57 and 30.88 min (entries 10, 79 and 80 in Table 2). This sequence is similar to that of the tripeptide Lys-Val-Lys, which was proved to exhibit a pro-collagen activity by Montanari and Guglielmo [34]. Besides, two tripeptide sequences very close to tripeptide sequences included in the pentadecapeptide from *Pyropia yezoensis* (…-Asp-Pro-Lys-… and …-Gln-Ala-Ile-…) were found in the peptides eluted at 23.03, 23.45 and 33.37 min (entries 43, 45 and 92 in Table 2): Asp-Pro-His and Gln-Ala-Ala, respectively. Indeed, the only variation in the sequences is located on the third amino acid, which is very similar in terms of property: another basic amino acid for the first one and another hydrophobic amino acid for the second one. However, despite these similarities, this does not mean that polypeptides or proteins exhibiting these sequences in the PROT-1 fraction are responsible for its pro-collagen activity, as biological functions of tripeptide sequences occurring in large peptide structures would not be necessarily the same as those of smaller peptides. Further studies involving the assessment of these particular peptides or proteins after purification are needed to understand their structure–function relationship.

The MMP-1 inhibition potential of the PROT-1 fraction was also evaluated but showed no significant inhibition (data not shown). In conclusion, the increase in collagen production induced by this fraction was probably not due to a decrease in the extracellular fibrillar collagen degradation directly associated with MMP-1 inhibition, contrary to what was shown by Ko et al. in their study of an *Ulva pertusa* protein extract [33]. The increase in collagen production might therefore be linked to the effect of the PROT-1 fraction on an intra-cellular mechanism, e.g., a biosynthesis pathway such as NF-kB or TGF-β/Smad signaling pathways, chaperone synthesis pathway (maintenance of conformation) or inhibition of MMP synthesis.

### 2.4. Effects of PROT-1 and H-PROT-1 on Hyaluronic Acid Production

Moisturization is the first step to fight against skin aging, by improving elastic properties of the skin [5]. A major component of the skin ECM, hyaluronic acid, plays a key role in skin moisturization by retaining water in the dermis, and thus lubricating the skin [39].

**Figure 9:**
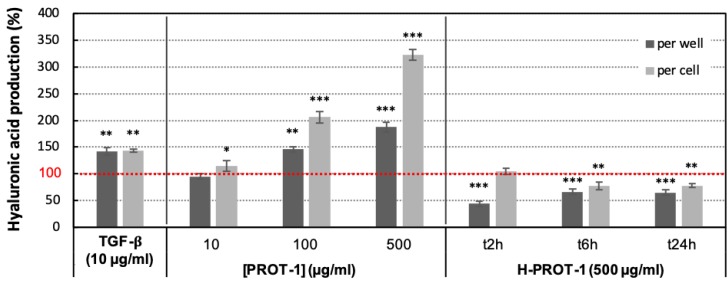
Hyaluronic acid production per well and per cell by the human dermal fibroblast cell line 1059. Results are expressed as the mean relative percentage of production, compared to the negative control (100%). Significant differences between values obtained with samples and negative control (*n* = 9) by * (*p* < 0.05), ** (*p* < 0.01) and *** (*p* < 0.001).

**Figure 10:**
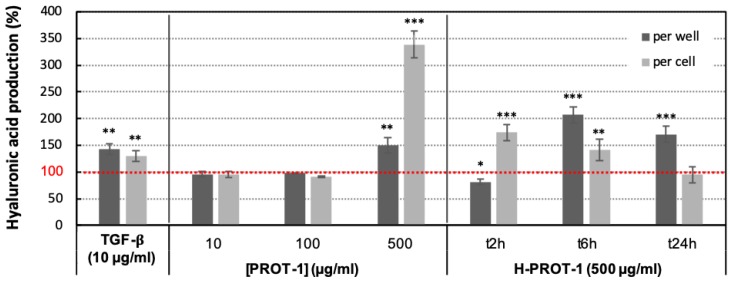
Hyaluronic acid production per well and per cell by the human dermal fibroblast cell line 1090. Results are expressed as the mean relative percentage of production, compared to the negative control (100%). Significant differences between values obtained with samples and negative control (*n* = 9) by * (*p* < 0.05), ** (*p* < 0.01) and *** (*p* < 0.001).

In this part of the study, the effects of PROT-1 and H-PROT-1 on the HA production by both the 1059 and 1090 fibroblast cell lines were investigated by an ELISA-like assay. TGF-β is known to stimulate HA production by dermal fibroblasts [40] and was then used as a positive control. Figure 9 and Figure 10 show that the HA production of both cell lines treated with the PROT-1 fraction was very significantly increased, compared to the negative control, and even to the positive control, TGF-β. More precisely, a TGF-β concentration of 10 µg/mL increased HA production per well and per cell of both cell lines, by about 45%, as PROT-1 induced a dose-dependent increase in hyaluronan production per well, especially with the 1059 cell line, reaching +46% and +87.3% at 100 and 500 µg/mL, respectively. However, unlike TGF-β, the PROT-1 fraction strongly and dose-dependently stimulated the HA production per cell, again mainly with the 1059 cell line; strong increases of 115%, 206% and 323% were indeed observed at 10, 100 and 500 μg/mL, respectively (Figure 9). The enzymatic hydrolysis of the PROT-1 fraction led to a loss of its pro-hyaluronic acid activity, which was very similar to what was previously observed for its pro-collagen activity. Indeed, the H-PROT-1 hydrolysates obtained after 2, 6 and 24 h induced a significant drop of HA production per cell of both cell lines at 500 μg/mL, more specifically a maximal reduction close to 245% after 24 h. This effect was particularly substantial for the 1059 cell line, which showed a strong and quick decrease in HA production. Indeed, the activity was 30% lower than that of the negative control after 6 h of hydrolysis. This loss of activity was slightly less significant with the 1090 cell line, as the HA production per cell was stimulated by the H-PROT-1 (t6h) fraction by about 42%. However, no stimulation could be observed at t24h. These results indicate that the native structure of the active proteins, which seems to be essential for their pro-collagen activity, is necessary for their pro-hyaluronic acid activity as well. They also confirm that fibroblast cell lines 1059 and 1090 are not equally stimulated by PROT-1 or H-PROT-1 fractions, pointing out that the physiological state of dermal fibroblasts might affect their stimulatory capacity.

Finally, TGF-β seemed to mainly enhance HA production by stimulating cell growth while the activity of PROT-1 fractions completely differed. The fact that these fractions highly promoted HA production per cell, compared to their effect on HA production per well, suggests that they would be able to directly induce HA biosynthesis by the fibroblasts rather than their growth. Fayat et al. showed that a brown seaweed aqueous extract from *Padina pavonica* inhibited the hyaluronidase [41]. This could be a way to further explore to better understand the mechanism of action involved in the pro-hyaluronic acid activity of the PROT-1 fraction.

## 3. Material and Methods

### 3.1. Material

All chemicals and reagents were purchased from Merck (Darmstadt, Germany). Fetal bovine serum, penicillin–streptomycin, trypsin–EDTA, and Eagle’s Minimum Essential Medium (EMEM) were purchased from Eurobio Ingen (Les Ulis, France). ELISA-like hyaluronic acid assay microplates were purchased from R&D systems (Wiesbaden, Germany). Human dermal fibroblasts (CCD-1059Sk, lot number 62062292, and CCD-1090Sk, lot number 204756) were obtained from ATCC Cell (Manassas, VA, USA). They were derived from the skin of a 20- and 46-year old woman, according to the provider’s information.

The green macroalga *Ulva intestinalis,* was cultivated and purchased from the aquaculture farm Algorythme on the Island of Ré (Ars-en-Ré, France). They were collected in summer 2017.

### 3.2. Methods

#### 3.2.1. Protein Extraction & SEPROSYS Extraction Procedure

First, 500 g of dehydrated *Ulva intestinalis* material was washed in 15 L of deionized water (1/30 (*w*/*w*)) for 10 min at room temperature (RT) and wrung with a fabric cone to remove as much water as possible (Scheme 1). The washed algae were then ground in 5 L of deionized water at 80 °C until obtaining 2-mm particles. The 5 L suspension of minced algae in water was transferred in a thermostated tank at 80 °C containing 2.5 L of deionized water. Extraction was processed under constant agitation with a bladed stirrer at a rotation speed of 10 spins/min for 2 h. The pulps were then removed from the tank and filtered with the fabric cone to collect the aqueous extract. Then, 13 L of extract was recovered and filtered with an ultrafiltration unit equipped with a 15 kDa Kerasep KBW membrane (Novasep Process, Pompey, France). Filtration was carried out at 80 °C at a pressure of 5 bars and a circulation flow of 450 L/h (circulation speed of 5 m/s) until obtaining a retentate around 4°B. Then, 1.5 L of retentate was demineralized by passage on a column containing 100 mL of Amberlite FPA 98, a strong anionic resin in the OH^−^ form, in series with a column containing 200 mL of Amberlite IR 120, a strong cationic resin in the H^+^ form. Circulation was processed with a peristaltic pump at a flow rate of 2 BV/h for the cationic resin. The deionized product was finally decanted in a water bath at 80 °C for 2 h (flocculation step). After centrifugation at 5000× *g* for 15 min at RT, the fraction, referred to as PROT-1, was neutralized to pH 7 with 1 M NaOH and lyophilized [22].

#### 3.2.2. Enzymatic Hydrolysis of PROT-1 Proteins

Five hundred milliliters of a 10 mg/mL solution of PROT-1 fraction was prepared in deionized water and heated at 50 °C, under magnetic agitation at 500 rpm. The protease preparation Flavourzyme^®^ was added at a ratio of 4% (*v*/*v*) and 10-mL aliquots were collected every hour for 24 h, and immediately heated at 90 °C for 20 min to inactivate the proteolytic enzymes. A control was also prepared without adding the protease preparation and similarly treated. Hydrolyzed samples, referred to as H-PROT-1 (t1h), (t2h), (t3h), (t4h), (t6h) and (t24h), were subsequently freeze-dried.

#### 3.2.3. Biochemical Composition

The neutral sugar content was determined according to the phenol-sulfuric method [42], using rhamnose as a standard; rhamnose was chosen as it is the main moiety of the parietal water-soluble polysaccharides of seaweeds from the genus *Ulva*, the so-called ulvans, which are the major polysaccharides extracted by water maceration of these seaweeds. The protein content of the dehydrated seaweed starting material was determined by the Kjeldahl method (N× 6.25) [43] while the protein content of the PROT-1 fraction was determined by the Lowry assay [44], using bovine serum albumin as a standard. The quantification method of polyphenols was adapted from the original one [45] using gallic acid as a standard; briefly, 50 µL of Folin–Ciocalteu reagent and 200 µL of 20% sodium carbonate were successively added to 100 µL of sample and the mixture was incubated in the dark for 45 min at RT, prior to absorbance reading at 730 nm. The ash content was determined by measuring the mass loss of samples heated for 15 h at 550 °C. The lipid content was measured by the method of Chabrol and Charonnat [46] using vegetal oil as a standard.

#### 3.2.4. High-Performance Liquid Size Exclusion Chromatography

The structural analysis and the molecular mass distribution of the proteins and peptides were assessed by high-performance liquid size exclusion chromatography (HPL-SEC), using a 1200 series HPLC system (Agilent Technologies), equipped with two size-exclusion chromatography columns in series: TSK gel 3000 SW and TSK gel 2500 PW (TOSOH Biosciences). The temperature of analysis was stabilized at 30 °C and 30 μL of extract or standard at 1 mg/mL was injected. The products were eluted with 20 mM phosphate buffer with 0.1% NaCl (pH 7) at a flow rate of 0.8 mL/min and detected at 210 nm. The standard curve was prepared using protein and peptide standards ranging between 1000 and 669,000 Da (thyroglobulin, 669 kDa; apoferritin, 443 kDa; β-amylase, 200 kDa; alcohol dehydrogenase, 150 kDa; albumin, 66 kDa, carbonic anhydrase, 29 kDa; α-lactalbumin, 14.2 kDa; cytochrome c, 12 kDa; insulin, 3 kDa; α-casomorphin 1-4, 1 kDa).

#### 3.2.5. Ultra-High-Performance Liquid Chromatography Coupled to High Resolution Mass Spectrometry Analysis (UHPLC-HRMS)

The total amino acid composition of the PROT-1 fraction was determined using an UHPLC system, “Acquity UPLC H-class”, (Waters, Milford, MA, USA) coupled to a HRMS system, “XEVO G2 S Q-TOF”, equipped with an electrospray ionization source (Waters, Manchester, England). The UHPLC system was formed by a quaternary pump (Quaternary Solvent Manager, Waters) and an automatic injector (Sample Manager-FTN, Waters) equipped with a 10 µL injection loop. Analyses were performed according to the UHPLC and MS parameters given in Table 3. The standard curve for each of the 20 essential amino acids was prepared using a standard solution at a concentration within the range 1–25 mg/L. The detection and quantification of total amino acids was performed after total acid hydrolysis of the sample. Briefly, 10 mg of the sample was solubilized in 1 mL of 1 M HCl and heated at 100 °C for two hours. After cooling at room temperature, the solution was neutralized by adding 1 M NaOH and filtered through a 0.22 µm filter prior to analysis, starting from a 2.5 mg/mL sample solution solubilized in water/methanol/formic acid 95:5:0.5 (*v*/*v*/*v*).

The sequence elucidation of peptides obtained in the H-PROT-1 (t1h) fraction was carried out after filtration of a 10 mg/mL sample aqueous solution on a 50 kDa membrane (Amicon Ultra 0.5 mL centrifugal filters, Merck) and analyzed using the same UHPLC-HRMS system. Analyses were performed according to the UHPLC and MS parameters given in Table 3.

The acquisitions and data processing were carried out using the Waters “Mass Lynx 4.1 version” software. The peptide sequences were checked using “Fragment ion calculator” software online (Institute for Systems Biology, Seatle, WA, USA), after the MS/MS fragmentation analysis.

#### 3.2.6. SDS-PAGE

SDS-PAGE was carried out using a Protean II xi cell electrophoresis unit from BIORAD (Hercules, CA, USA) with a stacking gel of 5% (*w*/*v*) and a separating gel of 17% (*w*/*v*) acrylamide in Tris-HCl 25 mM, pH 8.3, glycine 0.18 M and SDS 0.1% (*w*/*v*). The separation was performed at 75 mA for 2 h. The protein bands were stained by Coomasie brilliant blue. The size markers (10–250 kDa) were purchased from Precision Plus Protein^TM^ Standard, BIORAD (Hercules, CA, USA).

#### 3.2.7. Cell Culture

Cells were cultured in EMEM supplemented with 10% (*v*/*v*) fetal bovine serum and 1% (*v*/*v*) antibiotic solution (10,000 U/mL penicillin, 10 mg/mL streptomycin), used as the complete culture medium. Cell lines 1059 and 1090 were cultured in a temperature-controlled humidified incubator with 5% CO_2_ at 37 °C. The cells were grown in 75 cm^2^ ventilated Falcon culture flasks (BD Biosciences, Franklin Lakes, NJ, USA) and subcultured by trypsinization (0.05% (*w/v*) trypsin, PAN Biotech, Aidenbach, Germany). The culture medium was changed every two or three days. Cells were used between the third and ninth passages for the experiments.

#### 3.2.8. Cell Viability

The MTT assay was used according to the method described by Mosmann [47]. This is a colorimetric assay allowing assessing cell viability, based on the reduction of a yellow tetrazolium salt, 3-(4,5-dimethylthiazol-2-yl)-2,5-diphenyltetrazolium bromide (MTT), by viable cells. In the presence of mitochondrial succinate dehydrogenase found in active living cells, the tetrazolium ring of MTT is reduced and forms a violet product, formazan. The yellow solution becomes purple and the intensity of the purple coloration is proportional to the number of viable cells. The MTT test is used to determine cell growth and cell viability.

Briefly, cells were seeded in 96-well Falcon microplates (BD Biosciences, Franklin Lakes, NJ, USA) at 5 × 10^4^ cells/mL in 100 μL of complete culture medium and incubated for 24 h. The medium was then removed and 100 μL of PROT-1 or H-PROT-1 (at 10, 100 or 500 µg/mL) prepared in complete culture medium was added in the wells. After 48 h, 25 μL of MTT (5% (*w*/*v*) in PBS) was added in each well and the microplates were incubated for 4 h at 37 °C. The medium was then removed and 200 μL of dimethyl sulfoxide was added in each well. The microplates were then incubated for 10 min prior to absorbance reading at 550 nm using a Fluostar Omega microplate reader (BMG LABTECH, Ortenberg, Germany).

#### 3.2.9. LDH Assay

This method is based on the fact that, during cell death, the loss of the cell membrane integrity leads to the release of cytoplasmic enzymes, such as lactate dehydrogenase (LDH), into the extra-cellular medium. It measures the activity of LDH released by damaged cells in the cell supernatant, good marker of cell death [48].

LDH release was measured with a commercially available LDH assay kit (Cytotoxicity Detection Kit, Roche, France), following the supplier’s instructions. Briefly, LDH that is released in the cell environment reduces NAD^+^ into NADH and H^+^ through the oxidation of lactate into pyruvate. Thereafter, a catalyst (diaphorase) transfers H/H^+^ from NADH and H^+^ to a tetrazolium salt (iodonitrotetrazolium, INT), to form a red colored formazan salt. The absorbance of the red colored formazan salt produced was measured at 492 nm using a Fluostar Omega microplate reader (BMG LABTECH, Ortenberg, Germany).

#### 3.2.10. Collagen Quantification

NHDF were seeded at a density of 5 × 10^4^ cells/well in 24-well culture microplates in complete culture medium. After 24 h, the medium was removed and 500 µL of PROT-1 or H-PROT-1 (at 10, 100 or 500 µg/mL) or a negative or positive control in EMEM containing 1% (*v*/*v*) antibiotic solution was added in each well. The microplates were incubated for 48 h at 37 °C and the collagen production was then measured using the Sirius Red staining procedure [49]. Briefly, the medium was removed and the cells were washed twice with PBS and then fixed for 1 h with 1 mL of Bouin’s solution at RT. Following fixation, the Bouin’s solution was removed and cells were washed twice with deionized water. The cells were then stained with 1 mL of Sirius Red solution (0.5 g of Sirius Red 80 in 500 mL of saturated aqueous solution of picric acid) for 1 h under stirring at RT. Samples were then washed successively with deionized water and 0.01 M HCl to remove unbound dye. The bound dye was finally solubilized in 500 μL of 0.1 M NaOH for 1 h under stirring and absorbance was read at 550 nm using a Fluostar Omega microplate reader (BMG LABTECH, Ortenberg, Germany).

Results were then expressed as the mean relative percentage of collagen production, compared to the negative control, using the following equations:

(1) $$collagen\;production\;per\;well\;\left(\%\right)=\frac{{\left[collagen\right]}_{sample}}{{\left[collagen\right]}_{negative\;control}}\times 100$$

(2) $$collagen\;production\;per\;cell\;\left(\%\right)=\frac{collagen\;production\;per\;well\;\left(\%\right)}{cell\;viability\;\left(\%\right)}\times 100$$

#### 3.2.11. Hyaluronic Acid Quantification

Hyaluronic acid produced and secreted by fibroblasts in the extra-cellular medium was quantified using an ELISA-like hyaluronic acid assay plate (R&D Systems, Minneapolis, MN, USA). Briefly, cells were seeded at 5 × 10^4^ cells/mL in 100 μL of complete culture medium in 96-well Falcon microplates (BD Biosciences, Franklin Lakes, NJ, USA ) and cultured for 24 h. The medium was then removed and 150 μL of positive (10 μg/mL of TGF-β) or negative control prepared in incomplete culture medium, was added in the wells. After 48 h, the hyaluronic acid concentration in the supernatants was measured according to the manufacturer’s instructions.

Results were then expressed as the mean relative percentage of hyaluronic acid production, compared to the negative control, using the following equations:

(3) $$hyaluronic\;acid\;production\;per\;well\;\left(\%\right)=\frac{{\left[hyaluronic\;acid\right]}_{sample}}{{\left[hyaluronic\;acid\right]}_{negative\;control}}\times 100$$

(4) $$hyaluronic\;acid\;production\;per\;cell\;\left(\%\right)=\frac{hyaluronic\;acid\;production\;per\;well\;\left(\%\right)}{cell\;viability\;\left(\%\right)}\times 100$$

#### 3.2.12. Statistical Analysis

All data are presented as means ± standard deviations of at least triplicates. The student’s t-test (independent, two-sided) was used to determine significant differences between experimental and control samples, using Sigma Plot 12.5 (Systat Software Inc., San Jose, CA, USA).

## 4. Conclusions

In this study, a protein-rich fraction extracted from *Ulva intestinalis* (PROT-1) was produced using an acid- and solvent-free procedure. PROT-1 contained various sets of proteins. The major one exhibited a MW close to 25 kDa. We showed that the PROT-1 fraction significantly increased in vitro collagen and hyaluronic acid production by normal human dermal fibroblasts and that these increased productions were not due to an increase in cell number but rather to an activation of cell metabolism related to collagen and hyaluronic acid biosynthesis. We also concluded that the molecular weight significantly influenced this bioactivity. Indeed, H-PROT-1 fractions, which were the enzymatic hydrolysates of the PROT-1 fraction, had no effect on fibroblast proliferation and did not stimulate collagen and hyaluronic acid biosynthesis, compared to PROT-1. Moreover, the collagen and hyaluronic acid productions were decreased in presence of some H-PROT-1 fractions, proving the protein or polypeptide origin of the pro-collagen and pro-hyaluronic acid activities of the PROT-1 fraction.

This fraction could thus be of significant interest for skin care prevention or treatment as it significantly promotes in vitro collagen and hyaluronic acid biosynthesis by dermal fibroblasts without activating cell proliferation. In vitro assessment of these pro-collagen and pro-hyaluronic acid activities, as well as cytotoxicity, are a fundamental and essential step to develop ingredients with anti-aging potential. However, according to European Cosmetic guidelines, the evaluation of cosmetics should mix instrumental measurements from both in vitro and ex vivo/in vivo model systems [50]. To further envisage the commercial human application of the PROT-1 fraction, it would consequently have to undergo ex vivo and in vivo experiments. Firstly, noninvasive ex vivo assays on reconstructed human skin models could be performed, to measure percutaneous absorption, cell renewal and/or synthesis and degradation of the MEC and its constituents, collagens and hyaluronic acid in particular. Secondly, in vivo tests might be carried out on a panel of representative volunteers, starting from a neutral and stable formulation, to determine its tolerability in terms of cutaneous (patch test) or ocular irritation, the sensation felt after applying, and its effectiveness (measurement of mechanical properties of the skin such as density, firmness, elasticity and moisture; assessment of depth and volume of wrinkles; analysis of the epidermis microrelief).

## Figures and Tables

**Table 1:** Biochemical composition of the PROT-1 fraction.

Fraction	Ashes	Proteins	Neutral Sugars	Uronic Acids	Lipids	Polyphenols
	% (*w*/*w*_dry extract_)					
PROT-1	20	51	22	6	<1	<1

**Table 2:** Peptide sequences identified by UHPLC-HRMS in the H-PROT-1 (t1h) fraction.

Entry	Retention Time (min)	Ion	Experimental Monoisotopic Mass (Da)	Mass Accuracy (ppm)	Peptide or Amino Acid
1	2.58	[M + H]^+^	233.1130	0.86	VD
2	3.20	[M + H]^+^	508.1899	2.56	EGESS
3	3.81	[M + H]^+^	247.1284	2.02	VE
4	4.16	[M + H]^+^	246.1456	2.84	I/LN
5	5.65	[M + H]^+^	247.1289	2.02	I/LD
6	5.65	[M + H]^+^	247.1294	2.02	EV
7	6.67	[M + H]^+^	294.1545	2.72	I/L-hexose
8	8.21	[M + H]^+^	261.1452	0.77	I/LE
9	8.81	[M + H]^+^	261.1452	0.77	I/LE
10	9.57	[M + H]^+^	590.2798	2.88	ETVNQ
11	9.80	[M + H]^+^	404.2132	1.98	I/LSAN
12	10.40	[M + H]^+^	419.2135	0.00	TVAE
13	10.70	[M + H]^+^	449.2243	1.56	TEVT
14	10.93	[M + H]^+^	328.1394	0.61	F-hexose
15	11.32	[M + H]^+^	418.2293	0.96	I/LSAQ
16	11.52	[M + H]^+^	555.2410	0.18	TGYTN
17	11.52	[M + H]^+^	203.14	4.43	AI/L
18	11.83	[M + H]^+^	546.2520	0.18	DAI/LNN
19	12.46	[M + H]^+^	502.2625	1.00	AI/LNGQ
20	12.66	[M + H]^+^	295.1294	1.69	FE
21	12.87	[M + H]^+^	475.2515	0.84	AI/LSNA
22	13.41	[M + H]^+^	367.1506	0.27	W-hexose
23	14.27	[M + H]^+^	350.1740	1.43	I/LMS
24	14.48	[M + H]^+^	526.2163	4.18	SAYDA
25	14.48	[M + H]^+^	526.2163	4.18	* DADA
26	15.29	[M + H]^+^	440.1667	0.68	EEY
27	16.18	[M + H]^+^	403.2188	0.07	DVVA
28	16.65	[M + H]^+^	237.1241	3.08	AF
29	17.22	[M + H]^+^	205.0982	2.44	W
30	17.60	[M + H]^+^	229.1552	2.18	I/LP
31	18.01	[M + H]^+^	492.2662	0.61	SI/LTTA
32	19.09	[M + H]^+^	458.2607	0.59	I/LPQT
33	19.09	[M + H]^+^	458.2607	0.59	QPI/LT
34	19.98	[M + H]^+^	295.1659	2.03	I/LY
35	21.39	[M + H]^+^	302.2077	0.66	I/LI/LG
36	21.76	[M + H]^+^	416.2517	1.20	* GSI/LA
37	21.76	[M + H]^+^	416.2517	1.20	NVI/LA
38	22.01	[M + H]^+^	409.2078	1.00	VGAY
39	22.01	[M + H]^+^	409.2078	1.00	I/LNY
40	22.25	[M + H]^+^	552.2316	1.81	EAEFG
41	22.64	[M + H]^+^	411.7227	6.07	YTI/LDPP *
42	22.64	[M + H]^+^	295.1654	0.34	YI/L
43	23.03	[M + 2H]^2+^	720.3186	0.99	QAAEAAESGDFKSE
44	23.25	[M + 2H]^2+^	841.8622	-	* QSDWSEAEAAHS
45	23.45	[M + H]^+^	777.3397	0.40	NQAAEAAESGDESKF
46	23.65	[M + H]^+^	459.2179	3.64	EEYTFTK
47	23.65	[M + H]^+^	459.2179	3.64	KFTTYEE
48	23.65	[M + H]^+^	459.2179	3.64	KFYTTEE
49	23.85	[M + H]^+^	550.2878	1.13	(I/L)GTYP
50	24.12	[M + H]^+^	790.4323	2.21	TI/LATI/LSNA
51	24.32	[M + H]^+^	329.1500	1.28	YF
52	24.53	[M + H]^+^	373.2276	3.22	KDTVI/LI/LG
53	24.53	[M + 2H]^2+^	766.8573	-	* KDI/LI/LI/L *
54	25.18	[M + H]^+^	401.2756	0.72	VVAI/L
55	25.18	[M + H]^+^	600.3360	1.33	SPGGI/LGI/L
56	25.18	[M + H]^+^	600.3360	1.33	SNI/LPGI/L
57	25.58	[M + H]^+^	873.3639	1.57	(YG)YGDI/LDA
58	25.58	[M + H]^+^	873.3639	1.57	(YG)GI/LDYDA
59	25.58	[M + H]^+^	873.3639	1.57	(YG)DI/LGYDA
60	25.58	[M + H]^+^	873.3639	1.57	(YG)DYGI/LDA
61	25.58	[M + H]^+^	873.3639	1.57	(DI/L)GYGYDA
62	25.86	[M + H]^+^	687.2986	0.17	DI/LGYGY
63	26.07	[M + H]^+^	579.3143	0.98	NVI/LAY
64	26.07	[M + 2H]^2+^	590.7799	3.71	QEYENI/LI/LGSQ
65	26.07	[M + H]^+^	555.2821	1.31	YI/LYP
66	26.73	[M + H]^+^	431.2497	0.86	DI/LAI/L
67	27.04	[M + H]^+^	401.2756	0.72	I/LVGI/L
68	24.69	[M + 2H]^2+^	604.7886	7.87	DSTWI/LTTAI/LSN
69	28.26	[M + 2H]^2+^	566.2830	5.14	SG(Q/AG)QEI/LI/LDI/LE
70	28.26	[M + 2H]^2+^	566.2830	5.14	QSGQEI/LI/LDI/LE
71	28.26	[M + 2H]^2+^	566.2830	5.14	TNGQEI/LI/LDI/LE
72	28.46	[M + H]^+^	726.4041	1.13	EI/LSPPI/LA
73	28.46	[M + H]^+^	726.4041	1.13	ESI/LPPI/LA
74	28.46	[M + H]^+^	726.4041	1.13	DTI/LPAI/LP
75	30.26	[M + H]^+^	577.3329	2.72	AFI/LI/LN
76	30.47	[M + H]^+^	645.4180	4.34	TI/LI/LVI/LS
77	30.47	[M + H]^+^	645.4180	4.34	I/LI/LTVI/LS
78	30.47	[M + H]^+^	645.4180	4.34	VI/LTI/LI/LS
79	30.88	[M + H]^+^	938.4491	2.66	EI/LTDTVNF
80	30.88	[M + H]^+^	938.4491	2.66	ETI/LDTVNF
81	31.35	[M + 2H]^2+^	640.3069	4.08	EGESSI/LTTAI/LSNA
82	31.35	[M + 2H]^2+^	640.3069	4.08	E(SG/DG/TG)ESI/LTTAI/LSNA
83	31.89	[M + 2H]^2+^	616.3179	6.44	(AP)EI/LI/LTVDYNP
84	31.89	[M + 2H]^2+^	616.3179	6.44	PVVDI/LTVDYNP
85	32.13	[M + H]^+^	558.3867	0.95	I/LTVI/LI/L
86	32.50	[M + H]^+^	746.4453	0.74	PI/LI/LGI/LYA
87	32.70	[M + H]^+^	890.4997	-	* I/LTI/LP
88	32.70	[M + H]^+^	890.4997	-	* YEI/LP
89	32.70	[M + H]^+^	675.4078	0.25	I/LPGI/LI/LY
90	32.70	[M + H]^+^	675.4078	0.25	I/LPI/LGI/LY
91	33.17	[M + 2H]^2+^	634.3411	4.10	SI/LDI/LPHI/LPTQF
92	33.37	[M + 2H]^2+^	691.8536	3.45	SI/LDI/LDPHI/LPTQF

* undetermined amino acids; () undetermined sequence.

**Table 3:** Ultra-high-pressure liquid chromatography (UHPLC) and mass spectrometry (MS) parameters used to determine the total amino acid composition of the PROT-1 fraction and to elucidate the peptide sequences obtained in the H-PROT-1 (t1h) fraction.

	Total amino Acid Composition Determination	Elucidation of Peptide Sequences
**UHPLC Parameters**		
Column	Acquity UPLC HSST3 (150 mm × 2.1 mm × 1.7 μm) maintained at 25 °C	
Flow rate (μL.min^−1^)	0.3 mL·min^−1^	
Gradient used for the total amino acid composition determination: water/methanol 95:5 (*v*/*v*) (**A**)/water/methanol 50:50 (*v*/*v*) (**B**) + 0.5% (*v*/*v*) formic acid Gradient used for the elucidation of peptide sequences: water (**A**)/acetonitrile (**B**) + 0.1% (*v*/*v*) formic acid	0 min: 100% A; 2 min: 100% A; 7 min: 30% A; 8 min: 100% A; 13 min: 100% A.	0 min: 100% A; 2 min: 100% A; 2.5 min: 99% A; 3 min: 99% A; 3.5 min: 98% A; 4 min: 97% A; 5.5 min: 97% A; 8.5 min: 95% A; 10 min: 95% A; 15 min: 90% A; 18 min: 90% A; 22 min: 80% A; 27 min: 80% A; 30 min: 70% A; 34 min: 70% A; 36 min: 0% A; 38 min: 0% A; 40 min: 100% A; 45 min: 100% A.
Injection	5 μL (4 °C)	
**MS Parameters**		
Mode	ESI^+^ MS^E^, centroid: - Function 1, low energy, 5 eV - Function 2, high energy, ramping from 10 to 30 eV	ESI^+^ MS/MS DDA, centroid: - Transition from MS to MS/MS and MS/MS to MS when ion intensity becomes higher than 500,000/s and lower than 10,000/s (or after 10 s), respectively - Collision energy ramping from 15 to 35 eV
Source temperature	120 °C	
Desolvation temperature	500 °C	
Gas flow rate of the cone	50 L/h	
Desolvation gas flow rate	300 L/h	
Capillary voltage	3 kV	
Sampling cone voltage	35 V	
Source offset	80 V	
Acquisition mass range	50–1200 *m*/*z* (0.5 scans/s)	50–2500 *m*/*z* (0.5 scans/s)
Lock-mass	Leucine Enkephaline (MW = 555.62 Da,1 ng/μL) with a flow-rate of 5 μL/min	

## Abbreviations

RT	room temperature
ECM	extracellular matric
HA	hyaluronic acid
MMP	matrix metalloproteinase
TIMP	tissue inhibitor of metalloproteinase
SM	size markers
HPL-SEC	high-performance liquid size-exclusion chromatography
MTT	3-(4,5-dimethylthiazol-2-yl)-2,5-diphenyltetrazolium bromide
LDH	lactate dehydrogenase
TGF-β	transforming growth factor beta
AP-1	activator protein 1
NF-κB	nuclear factor-kappa B
EDTA	ethylenediaminetetraacetic acid
